# Emphysematous cholecystitis. Advantages of abdominal ultrasound in the ED

**DOI:** 10.1186/2036-7902-6-S1-A7

**Published:** 2014-01-31

**Authors:** M Algaba-Montes, A Oviedo-García, D Nuñez-Hospital, J Lopez-Libano, JM Alvarez-Franco, N Diaz-Rodriguez, A Rodriguez-Lorenzo

**Affiliations:** 1 Emergency Department, Valme Hospital, Seville, Members of the Working Group of Ultrasound SEMES_Andalucía and SEMERGEN, Spain; 2 Emergency Department, Valme Hospital. Seville, Spain; 3 Critical Care Department, Miramar Hospital, Mallorca, Member of the Working Group of Ultrasound SEMERGEN, Spain; 4 Emergency Department, IB-Salut, Ibiza, Member of the Working Group of Ultrasound SEMERGEN, Spain; 5 Primary Care. Barbadás Primary Care Center. Ourense, Member of the Working Group of Ultrasound SEMERGEN, Spain; 6 Radiology Department. Perpetuo Socorro Hospital, Vigo, Member of the Working Group of Ultrasound SEMERGEN, Spain

## Background

Emphysematous cholecystitis (EC) is an entity with high morbidity and mortality, and therefore require a diagnosis agile and dynamic, allowing appropriate management to avoid complications. The emergency ultrasound (US) allows a versatile and comprehensive management, improving the prognosis of this disease in the majority of cases.

## Objective

we present a case of EC, diagnosed at ER, through the use of US scanning used by Emergency Phisicians.

## Patients and methods

a patient with abdominal pain, with a final diagnosis of an EC assessing US, performed by EP.

## Results

We report the case of a 72 year old patient with prior stroke without sequelae and hypertensive, with abdominal pain of 7 days duration, high fever and bilious vomiting. Physical examination was marked hypotension (80/45 mmHg), distal coldness, pallor and sweating, 38.5 ° C, 145 spm. The distended abdomen with abolished peristalsis and positive Murphy right upper quadrant. Rest without findings of interest. Analytically glucose was 505 mg / dl, creatinine of 2.44 mg / dl, bilirubin 2.2 mg / dl, AST 350, LDH 407, amylase 125, 19500 leukos with neutrophilia and pH of 7.13, with lactic 12. Was performed in consultation abdominal US showed a thickened gallbladder wall (8 mm), well-circumscribed, oval, distended and gas in the same light, compatible with emphysematous cholecystitis. Support measures were initiated, antibiotics, insulin therapy and emergency surgery was indicated. This allowed a favorable high after joining UCI in 7 days without further complications.

## Conclusion

EC is a rare entity that represents 1% of all cholecystitis, clinically indistinguishable, but with a worse prognosis (25% mortality) and more complications. Here debut comes as poorly controlled diabetes. The use of abdominal US in ER allows for both a rapid and versatile, with proper treatment start, this being vital to good patient outcomes.
